# Death of Eminent Men

**Published:** 1855-01

**Authors:** 


### DEATH OF EMINENT MEN.

Dr. GOLDING BIRD, so well known by his treatise on *Urinary Deposits*, died in October last, of a malady, the elucidation of which has rendered his name distinguished among the cultivators of medical science. He died of kidney disease, in the 38th year of his age.

Prof. LALLEMAND, author of a well known treatise on *Spermatorrhea*, died recently at Marseilles.

Prof. EDWARD FORBES, one of the most eminent naturalists of the day, died at Edinburgh, on the 16th of November, from the effects of a chronic disease, aggravated by exposure on a geological excursion. It is only a few months since he was elected to the Chair of Natural History in the University of Edinburgh.

